# Reducing Alaska Native paediatric oral health disparities: a systematic review of oral health interventions and a case study on multilevel strategies to reduce sugar-sweetened beverage intake

**DOI:** 10.3402/ijch.v72i0.21066

**Published:** 2013-08-05

**Authors:** Donald L. Chi

**Affiliations:** University of Washington, School of Dentistry, Department of Oral Health Sciences, Seattle, Washington, USA

**Keywords:** oral health disparities, Alaska Native health disparities, dental caries prevention, children, dental workforce, primary intervention in oral health, sugar-sweetened beverages

## Abstract

**Background:**

Tooth decay is the most common paediatric disease and there is a serious paediatric tooth decay epidemic in Alaska Native communities. When untreated, tooth decay can lead to pain, infection, systemic health problems, hospitalisations and in rare cases death, as well as school absenteeism, poor grades and low quality-of-life. The extent to which population-based oral health interventions have been conducted in Alaska Native paediatric populations is unknown.

**Objective:**

To conduct a systematic review of oral health interventions aimed at Alaska Native children below age 18 and to present a case study and conceptual model on multilevel intervention strategies aimed at reducing sugar-sweetened beverage (SSB) intake among Alaska Native children.

**Design:**

Based on the Preferred Reporting Items for Systematic reviews and Meta-Analyses (PRISMA) Statement, the terms “Alaska Native”, “children” and “oral health” were used to search Medline, Embase, Web of Science, GoogleScholar and health foundation websites (1970–2012) for relevant clinical trials and evaluation studies.

**Results:**

Eighty-five studies were found in Medline, Embase and Web of Science databases and there were 663 hits in GoogleScholar. A total of 9 publications were included in the qualitative review. These publications describe 3 interventions that focused on: reducing paediatric tooth decay by educating families and communities; providing dental chemotherapeutics to pregnant women; and training mid-level dental care providers. While these approaches have the potential to improve the oral health of Alaska Native children, there are unique challenges regarding intervention acceptability, reach and sustainability. A case study and conceptual model are presented on multilevel strategies to reduce SSB intake among Alaska Native children.

**Conclusions:**

Few oral health interventions have been tested within Alaska Native communities. Community-centred multilevel interventions are promising approaches to improve the oral and systemic health of Alaska Native children. Future investigators should evaluate the feasibility of implementing multilevel interventions and policies within Alaska Native communities as a way to reduce children's health disparities.

Tooth decay (also known as dental caries) is the most common paediatric disease in the United States (1). It is an infectious disease caused by bacteria that metabolise ingested carbohydrates and release acid by-products that demineralise tooth structure. Frequent consumption of carbohydrates and insufficient exposure to topical fluorides (found in toothpastes, fluoridated drinking water and fluoride treatments received during dental visits) results in tooth decay. When left untreated, tooth decay leads to pain, infection, hospitalisation and in rare cases death (2). The consequences of tooth decay during childhood manifest throughout adolescence and adulthood and can result in negative social outcomes, including school absenteeism, poor grades and low quality-of-life (3, 4). Collectively, these factors underscore the importance of preventing tooth decay.

Tooth decay affects Alaska Native children and adolescents of all ages. A 1987 study reported that the prevalence of baby bottle tooth decay (BBTD) (defined as decay on 2 or more primary maxillary incisors) ranged from 44–85% for 3- to 5-year-old Alaska Native children (compared to 5–11% in non-Alaska Native paediatric populations) (5). Two studies from the early 1990s reported that the mean number of decay, missing or filled surfaces (dmfs) for children aged 3–5 in the Alaska Head Start Program was 7.9 for all children and 11.0 for children of Alaska Native descent (6, 7). Nearly 66% of Alaska Native pre-schoolers presented with untreated tooth decay compared to 19% of non-Alaska Native pre-schoolers (6). Tooth decay rates were highest for Alaska Native children living in rural areas (rural=12.1 dmfs vs. urban=6.1 dmfs) (7) and in Alaska's Yukon-Kuskokwim Delta region (12.1 dmfs) (6). Older children and adolescents also have high tooth decay rates (8, 9). More recent studies suggest that the paediatric tooth decay epidemic in Alaska Native communities has not abated (10–12).

Historically, tooth decay was not a public health problem in Alaska Native communities because traditional diets were low in fermentable carbohydrates (13). This changed with the introduction of a cash-based economy and Western-style diets high in refined sugars. Over time, highways and air travel made it easier to transport foods and beverages to Alaska Native communities and local demand for Western consumables increased. In addition to dietary changes, many Alaskan Natives do not have access to fluoridated community water, which fuelled the tooth decay epidemic. Furthermore, Alaska Native communities are sparsely scattered throughout Alaska and many of these communities are only accessible by air, which makes dental care delivery logistically difficult and expensive. Dentist shortages constrain the supply of dental providers in isolated communities. Children requiring oral rehabilitation under general anaesthesia are flown with family members to regional health centres within Alaska or as far away as Anchorage, Juneau and Seattle for treatment provided in hospital operating rooms (14).

The magnitude of the tooth decay crisis is illustrated by the 2011 American Academy of Pediatrics policy statement on early childhood caries in Alaska Native and American Indian communities, which calls for community-based research on methods to prevent and manage tooth decay (15). However, the extent to which population-based oral health interventions have been implemented in Alaska Native paediatric populations is unknown. The 2 goals of this study are: (a) to conduct a qualitative systematic review on population-based interventions aimed at improving the oral health of Alaska Native children and adolescents and (b) to present a case study that provides readers with a conceptual model on multilevel oral health interventions. The case study focuses on strategies to reduce sugar-sweetened beverage (SSB) intake – one of the main risk factors of tooth decay. The conceptual model presented can be used to develop and test future multilevel interventions and policies aimed at reducing oral health disparities and improving the oral and systemic health of Alaska Native children.

## Methods

### Eligibility criteria

This review focused on clinical trials, evaluation studies, systematic reviews, conference abstracts and reports related to oral health interventions focusing on Alaska Natives below age 18. The review included studies published in English in calendar years 1970–2012. Editorials and commentaries were excluded.

### Protocol registration and information sources

There was no registered protocol for this study. Four online databases (Medline, Embase, Web of Science and GoogleScholar) were used to identify relevant references and citations using the search terms “Alaska Native”, “children” and “oral health”. The last search date was October 5, 2012. Health foundation websites (e.g. Kellogg Foundation, Robert Wood Johnson Foundation) were visited to identify reports.

### Search strategy, data collection and reporting of data

**Fig. 1:**
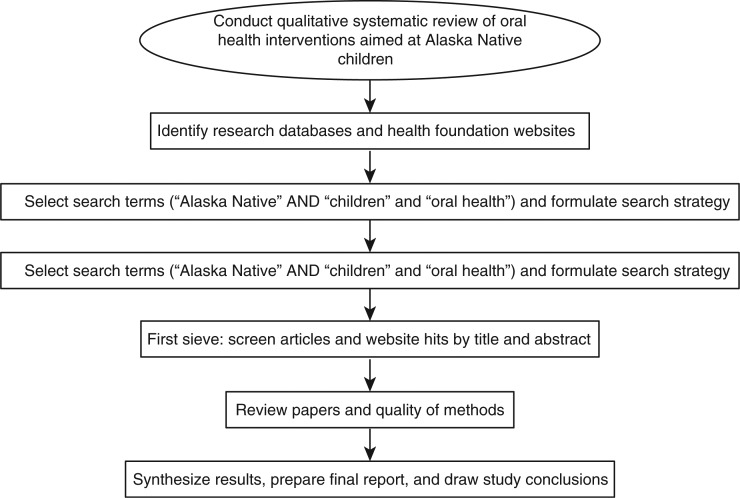
Flow chart indicating search strategy for qualitative systemic review on oral health interventions for Alaska Native children.

The search strategy, based on the Preferred Reporting Items for Systematic reviews and Meta-Analyses (PRISMA) statement (16), is summarised in Fig. 1. The PubMed advanced search builder (i.e. Medline) was accessed on October 5, 2012. The terms “Alaska Native” AND “children” AND “oral health” were entered into the search fields. Identical methods were used in Embase, Web of Science and GoogleScholar. All returned hits in GoogleScholar were evaluated for relevance by following individual links. PICO (patient/population, intervention, comparison group and outcome) criteria were used to screen studies and the following data were obtained: level at which the intervention was targeted (e.g. community, caregiver, patient), follow-up period, intervention effectiveness and challenges encountered. Study limitations were noted. This was a qualitative systematic review and no summary measures were calculated.

### Assessment of bias

Risk of bias within studies was assessed at the study-level (adequacy of allocation concealment) and at the outcome-level (reliability and validity). The risk of bias across studies was assessed by examining the degree to which there was selective reporting within studies.

## Results

### Study selection

**Fig. 2:**
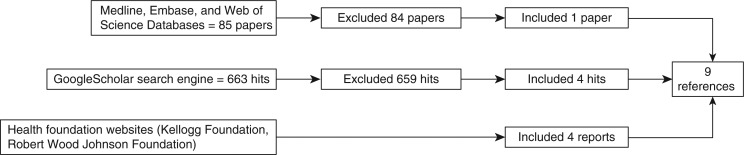
Flow chart indicating number of references identified and included in final qualitative systemic review on oral health interventions for Alaska Native children.

A total of 85 studies from Medline, Embase and Web of Science databases were screened and 33 were excluded based on the study title (Fig. 2). Of the remaining 52 studies, 1 study met the eligibility criteria. Of the 663 GoogleScholar hits screened, 658 hits were excluded based on information provided in the title and 1 hit was excluded because it was a duplicate from the previous database search, leaving 4 hits that met the eligibility criteria. Four reports were identified from health foundation websites. A total of 9 publications (17–25) met the inclusion criteria and were included in the qualitative review.

### Study characteristics

The data abstract from each study are summarised in Table I. The 9 publications were organised into 1 of the 3 categories: caregiver and community education (2 publications); dental chemotherapeutics (2 publications); and mid-level dental providers (5 studies). The 2 education publications were from the same prospective study from the 1990s. The 2 dental chemotherapeutic publications were from the same prospective xylitol gum trial that was discontinued. Of the 5 retrospective mid-level provider publications, 4 focused on dental health aide therapists (DHATs) and one was a simulation study of primary dental health aides (PDHAs).

**Table I:** Data abstracted from studies included in qualitative systemic review of oral health interventions for Alaska Native children (n =9 studies)

Study category	References	Patient/population	Intervention	Level of intervention	Comparison groups	Outcome	Follow-up period	Challenges	Study limitations
Reducing tooth decay in Head Start children through community and caregiver education	Bruerd & Jones (17)	Alaska Native and American Indian Head Start children (12 study sites)	Oral health education for lay community volunteers, health workers, and site coordinators; educational materials and counselling for caregivers Media campaign on oral health	Provider, Caregiver, Community	Ongoing Baby Bottle Tooth Decay (BBTD) Prevention Program from 1986 to 1994 BBTD Prevention Program discontinued in 1989	Percent reduction in the prevalence of children with BBTD (defined as decay of 2 primary maxillary anterior teeth) Significant decreases in BBTD for communities with ongoing BBTD Prevention Programs but not within communities that discontinued the BBTD Prevention Program	8 years	Programme discontinuation most often because of staff turnover Importance of recruiting study staff members from local population Difficulty of institutionalising pilot programmes into organisation New strategies needed to reach highest risk children who change residences frequently and do not access health care services	Small number of communities that may not be representative of Alaska Native and American Indian communities The absence of tooth-level interventions (e.g. fluoride varnish)
	Bruerd et al. (18)	Alaska Native and American Indian Head Start children (12 study sites)	Oral health education for lay community volunteers, health workers, and site coordinators; Educational materials and counselling for caregivers Media campaign on oral health	Provider, Caregiver, Community	High intensity (community volunteers and site coordinators trained directly by study team) Medium intensity (site coordinators trained by study team at a central location) Low intensity (site coordinators received no training)	Percent reduction in the prevalence of children with BBTD (defined as decay of two primary maxillary anterior teeth) Significant decreases in BBTD within comparison groups and for all 12 study sites	5 years (interim results at year 3 reported)	20% increase in BBTD in the single Alaska Native community (one of the high intensity groups) Waning enthusiasm, personnel loss, lack of follow through on study protocols Limited fidelity monitoring Resources involved in assembling community planning group	No operationalised definition of BBTD (obtained from Bruerd and Jones (17)) Unable to identify intervention components that were most important Small number of communities that may not be representative of Alaska Native and American Indian communities
Dental chemotherapeutics for pregnant women	Jolles et al. (19)	Pregnant Alaska Native women in the Yukon-Kuskokwim Delta	Xylitol chewing gum	Caregiver	Xylitol chewing gum Placebo chewing gum	Percent of children with dental caries	None	Difficulties recruiting and retaining study participants Lack of engaging and attractive advertising within communities about study Lack of face-to-face interaction with community members Lack of local community-based study personnel Lack of input and involvement from participating communities	No outcomes assessed. Trial was discontinued because of problems recruiting and retaining study participants
	Riedy (20)	Pregnant Alaska Native women in the Yukon-Kuskokwim Delta	Xylitol chewing gum	Caregiver	Xylitol chewing gum Placebo chewing gum	Percent of children with dental caries	None	Distrust of researchers Difficulties in accessing geographically isolated communities Problems with travelling to communities because of inclement weather Patient acceptability of dental treatments and chemotherapeutics, especially during pregnancy Low cultural acceptability of gum chewing during lactation Difficulties recruiting participants within study period High cost of conducting research in rural Alaska	No outcomes assessed. Trial was discontinued because of problems recruiting and retaining study participants
Increasing access to dental care by training mid-level providers	Fiset (21)	Alaska DHATs	DHATs	Patient/provider	No comparison group	Record keeping Cavity preparation Cavity restoration Patient management Patient safety	None	DHATs unlikely to solve access to dental care problems in rural Alaska	Single examiner
	Bolin (22)	Alaska DHATs	DHATs	Patient/provider	DHATs Dentists	Clinical technical performance	None	Clinical technical performance one of many measures of quality of care	Single examiner; no intra-rater reliability data provided Control selection protocol unclear
	RTI International (23)	Alaska DHATs	DHATs	Patient/provider	DHATs Dentists	Patient satisfaction, oral health-related quality of life, and perceived access to dental care Oral health status Clinical technical performance Implementation of community-based preventive programmes	None	Difficulties recruiting and retaining qualified DHATs DHATs spend bulk of time treating patient with less emphasis on community-based preventive programmes	Too few DHATs to evaluate effect of DHATs on access to dental care Cross-sectional study design
	Bader et al. (24)	Alaska DHATs	DHATs	Patient/provider	DHATs Dentists	Clinical technical performance	None	Clinical technical performance one of many measures of quality of care	Small number of restorations evaluated
	Kiley et al. (25)	Alaska PDHAs	PDHAs	Patient/provider	Pre-PDHAs PDHAs PDHAs+intensive staff model PDHAs+additional prevention	Simulation model to compare PDHAs versus alternative service delivery models on oral health outcomes (dental utilisation, various measures of tooth decay)	None	PHDAs focus on prevention and outreach, but most communities are in need of providers who can deliver restorative care Inherent tension between improving oral health outcomes for children and adults given limited resources	Selective reporting of oral health outcomes measures The PHDAs+additional prevention model may not be acceptable to pregnant women (no evaluation of patient acceptability of alternative interventions) No evaluation of costs

### Study results

Two publications described results from a prospective community-based trial on educating lay community volunteers, health workers and site coordinators to counsel caregivers of Head Start enrollees on strategies to reduce BBTD (17, 18). In addition, there was a community-level media campaign promoting tooth decay prevention in children. In the first study (17), there were 12 Alaska Native and American Indian Head Start study sites (1 in Alaska and 11 in the Continental US). The authors compared pre- and 3-year post-intervention BBTD rates. Study sites were classified as high, medium or low intensity depending on the type of training received by community members. Overall, there were significant reductions in BBTD (25%; p<0.001) as well as within the 3 intervention groups: high (33%; p<0.001), medium (18%; p<0.001) and low (27%; p<0.001). However, there were increases in BBTD in 3 sites, including a 20% increase in the Alaska Native site (1 of the 4 high-intensity sites). This study suggests that community-based interventions can reduce tooth decay in Head Start children, but that subgroups of high-risk children may require more intensive interventions.

In the second study (18), the authors compared BBTD rates for Head Start children in the sites that maintained a prevention programme (1986–1994) and those that stopped the prevention programme in 1989. While there was a significant decrease in the overall tooth decay rate across all 12 sites (25%; p<0.001), stratified analyses indicated statistically significant decreases only for the sites that maintained a prevention programme (28%; p<0.001) compared to those that stopped (15%; p>0.05). These findings suggest that preventive oral health programmes require on-going funding and structural support.

Two publications described the challenges and lessons learned from a xylitol chewing gum trial aimed at pregnant Alaska Native women in the Yukon-Kuskokwim Delta (19, 20). The proposed study was a randomised control trial comparing infant caries rates for pregnant women receiving a dental chemotherapeutic (chlorhexidine and xylitol chewing gum) and women who received a placebo intervention. The researchers conducted preliminary research prior to the intervention, but the trial was discontinued because of difficulties in recruiting and retaining study participants. In a conference abstract, researchers reported findings from qualitative work that examined investigator-related reasons for the discontinuation of the trial (19). These reasons included unattractive and non-engaging advertising of the trial during recruitment, limited investigator interaction with community members and the absence of local study managers. A second study noted additional challenges including community distrust of researchers, pregnancy as an inappropriate time to introduce unfamiliar chemotherapeutics and dental treatment (both of which were perceived as unsafe), cultural beliefs discouraging gum chewing during pregnancy and lactation and difficulties in recruiting women before delivery without input from other family and community members (20). Findings from these studies indicate that intervention research in Alaska Native communities requires sufficient pre-intervention work.

There were 5 studies that evaluated workforce interventions aimed at training mid-level dental care providers. Three studies examined tooth- and patient-level outcomes associated with dental treatment provided by dental health aide therapists (DHATs) in Alaska Native communities (21–24). The first evaluation was a qualitative examination of a cohort of newly trained DHATs who had been providing dental care for 9 months (21). The 4 practising DHATs met Indian Health Service guidelines on recording keeping and met standards of care regarding cavity preparation and restoration, patient management and patient safety. The second and third studies also examined the quality of dental care provided by DHATs and adopted the following measures: patient satisfaction, technical performance (composite and amalgam restoration preparation and placement, stainless steel crown preparation and placement) and dental record quality (organisation, completeness, appropriateness of treatment plan, up-to-date bitewing radiographs) (23, 24). More than 90% of caregivers of children aged 6–17 reported being “usually” or “always” satisfied with the care provided by a DHAT (23). None of the composite preparations completed by DHATs were classified as deficient and similar proportions of composite restorations placed by DHATs versus dentists were deficient (15 and 12%, respectively) (23, 24). Two of the 13 amalgam preparations completed by DHATs were classified as deficient and lower proportions of amalgam restorations placed by DHATs versus dentists were deficient (12 and 22%, respectively) (23, 24). No deficiencies were found for stainless steel crown preparations and a lower proportion of DHATs had deficient stainless steel crown placement compared to dentists (3 and 9%, respectively) (23, 24). Record keeping by DHATs was consistent with evaluation criteria (23). The fourth study arrived at similar conclusions regarding quality of dental treatment (22). There was no significant difference between DHATs and dentists in consistency of diagnosis or postoperative complications (22). Overall, patients were highly satisfied, technical performance of DHATs was comparable to dentists, and recording keeping was of high quality.

A fifth study was part of a master's degree thesis involving simulation models of PDHAs in Alaska (25). PDHAs are prevention-oriented health providers who work under the general supervision of dentists. They apply fluoride and sealants in school-based oral health promotion programmes. The study compared a baseline “pre-intervention” scenario with 3 alternative models of care: (a) the PHDA program; (b) the PHDA program with an intensive staffing model (high productivity staff model with shorter dental appointments and longer staff hours) and (c) the PHDA program with robust prevention (xylitol gum and chlorhexidine rinse for children and pregnant mothers) and early implementation of preventive oral health promotion programmes. Simulations were projected over 20 years and outcome measures included: percentage of children receiving clinic-based dental care, proportion of children receiving school-based oral health promotion services, mean number of decayed, missing and filled teeth (DMFT), percentage of tooth decay-free children and significant caries index (SiC index, defined as the mean DMFT in the one-third of children with the highest DMFT). Compared to baseline, the PDHA program was projected to increase dental care utilisation by 15% (from 75 to 90%) (25). More than 90% of children would receive school-based oral health promotion services (up from 53%). The mean DMFT for children below 5 years would decrease (from 7.4 to 4.9). The SiC index would decrease 50% (from 12.3 to 6.1) for children and 70% for 12-year-olds (10.5–3.3). The robust prevention model would result in more than 50% tooth decay-free 5- to 18-year-olds, compared to 8% in the PDHA model or PDHA/intensive staffing model and 0% in the baseline model. Based on these simulation models, it appears that the PDHA/robust prevention model results in improved paediatric oral health outcomes.

### Assessment of bias

It was not clear from the 2 prospective intervention studies that allocation was concealed (i.e. whether the participating sites knew which intervention group they had been assigned to) (17, 18). Intervention concealment was unlikely in this non-randomised community-level intervention, which could have introduced bias in outcome assessment. The authors note that intervention “contamination” was a possibility (e.g. if a site knew that it had received medium-or low-intensity training rather than high-intensity training then the site may have taken extra steps to train providers), which could lead to misclassification bias on the main predictor variable (degree of intervention intensity). In addition, there were no reliability or validity data reported. Based on these factors, there is high risk of bias in these studies. For the 4 DHAT evaluations, either there was no control group (21) or it was unclear how preparations and restorations completed by dentists (controls) were selected (22–24). Intra- and inter-examiner reliability data were not presented. Based on these factors, there is medium risk of bias in these studies. Bias was not a concern for the 2 studies describing a discontinued intervention (19, 20).

## Discussion

Few population-based oral health interventions have been conducted in Alaska Native paediatric populations. A community-based educational intervention from the 1980s is the main documented intervention aimed at addressing tooth decay in Head Start pre-schoolers, but the intervention did not reduce tooth decay in Alaska Native children. A more recent intervention sought to improve Alaska Native paediatric oral health disparities by preventing dental disease in pregnant women, but this intervention was discontinued because of difficulties in recruiting and retaining study participants. The only existing intervention in Alaska is the training of mid-level dental providers, but it is too early to conclude whether mid-level providers will meaningfully improve the oral health of Alaska Native children. There are 3 observations that can be drawn from these studies.

The first observation is that there is insufficient research on Alaska Native paediatric oral health disparities, which is a concern given the extent and severity of the Alaska Native tooth decay epidemic. While a number of studies focus on the oral health of American Indian children in the continental US (26, 27), there are fewer observational studies and no randomised clinical trials on Alaska Native children. There are 3 possible reasons. The first is the geographic isolation of Alaska, which makes research in Alaska Native communities challenging. Identifying and retaining local research staff in small communities can be difficult. Seasonal variations in weather affect air- and land-based travel and restrict field research. In addition, local accommodations are limited and costly. The second is the substantial time commitment required to initiate research within Alaska Native communities. Successful community-based participatory research is based on cooperation and mutual trust, both of which are developed over time through face-to-face contact. Federal grant timelines may not align with local processes and research protocols can require substantial time to obtain proper approvals. The third is the assumption that the determinants of oral health disparities in Alaska Native and American Indian communities are the same, which leads to the belief that research conducted in American Indian communities is generalisable to Alaska Native communities. While indigenous communities have shared characteristics (e.g. history of discrimination, high levels of poverty and disease, social isolation from population centres), there are cultural, social, behavioural and linguistic traits that differentiate Alaska Natives from other indigenous groups. These differences are relevant in developing culturally sensitive interventions to improve the oral health of Alaska Native children.

The second observation is that recent oral health-related interventions have focused mostly on provider-level solutions. Mid-level dental providers may help to address workforce shortages and unmet treatment needs in isolated Alaska Native communities, especially for Alaska Native children (22). However, DHATs practice in Alaska Native communities with high levels of dental disease, which may initially result in the adoption of a surgical model of care that replicates the existing dental care delivery system and does not address the aetiology of dental disease. While it is less expensive to train DHATs than to train dentists, surgical approaches place tremendous strain on state dental Medicaid program budgets and are unsustainable in light of scare financial and human resources. It is too early to assess whether DHATs will have a long-term effect on paediatric tooth decay rates. However, oral disease prevention is one of the key components of the current DHAT curriculum, which has the potential to translate into meaningful community-based preventive activities. There is a need to ensure that DHATs devote meaningful efforts to community-based prevention and to evaluate community- and patient-level outcomes associated with such efforts. Provider-level solutions must be coupled with intensive prevention-oriented strategies that are evaluated for demonstrable efficacy and effectiveness.

The third observation is related to the first 2 observations: the absence of multilevel oral health interventions. Multilevel interventions address “the individual patient as well as 2 levels of contextual influence” (28). Levels of contextual influence beyond the individual patient include: families, health providers, health organisation settings, local community environment, state health policy environment and national health policy environment (28). Thus, multilevel interventions in dentistry should: (a) seek change at 3 or more levels; (b) employ chemotherapeutic, behavioural and/or social intervention strategies; and (c) be implemented in various settings (e.g. individuals, families, communities). Because tooth decay is a complex and multifactorial disease, single-level interventions that focus entirely on increasing the numbers of providers or dental utilisation rates are unlikely to meaningfully address oral health disparities. There are a number of strategies for combining interventions at different levels (e.g. accumulation, amplification, facilitation, cascade, convergence) (29). These approaches have not yet been empirically evaluated in oral health.

**Fig. 3:**
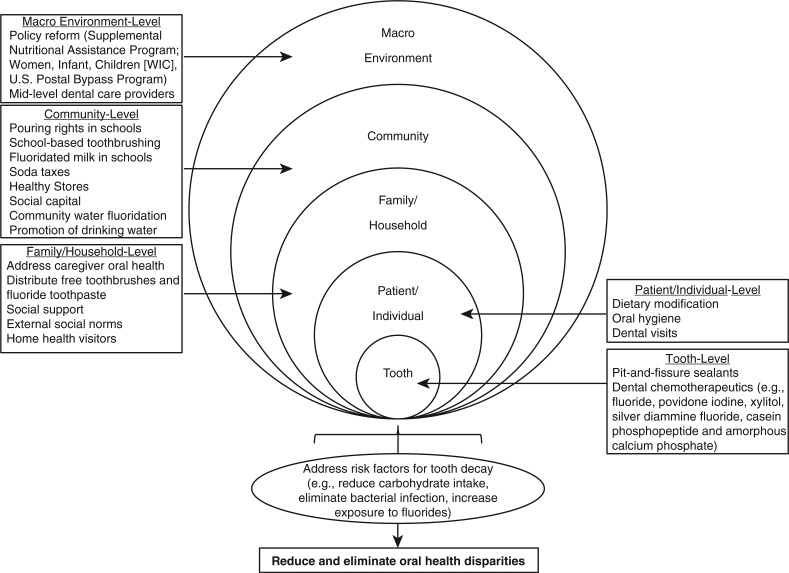
Conceptual model of potential multilevel strategies to address risk factors for tooth decay and reduce Alaska Native children's oral health disparities.

To provide readers with an example of how we can use findings from the systematic review to develop multilevel oral health interventions, a case study is presented on strategies to address SSB intake, one of the major risk factors for tooth decay in Alaska Native children and adolescents. The conceptual framework of the case study (Fig. 3) is based on the multilevel influence model presented by Taplin and colleagues (28) and the sociocultural oral health disparities model proposed by Patrick and colleagues (30), which posits that the determinants of oral health disparities within vulnerable subgroups are multifactorial. The case study is not meant to be exhaustive. Rather, it serves as a starting point for investigators interested in developing comprehensive multilevel strategies aimed at improving the oral health of Alaska Native children.

### Case study: multilevel strategies to reduce sugar-sweetened beverage intake

#### Background and overview

Health providers and public health officials in rural Alaska have expressed concerns about the health consequences of SSB intake among Alaska Native children. Most SSBs contain high fructose corn syrup, which is fermentable by oral bacteria, and carbonated SSBs erode tooth surfaces. SSB intake is associated with a number of preventable diseases, including tooth decay, obesity and type II diabetes (31–35). Children who use professional dental care services may receive preventive care and some form of dietary advice about the oral health consequences associated with SSBs. Such approaches are based on tooth-level chemotherapeutic interventions (e.g. application of topical fluoride varnish) and well-meaning but ineffective attempts to change patient-level behaviours by dispensing generic advice about the need to eliminate SSBs from the diet. Current patient-level behavioural approaches employed by dental providers are likely to be paternalistic, generic (i.e. not tailored to patient's current knowledge, practices or social conditions), and not rooted in behaviour change theory. Such approaches do not account for the family-, school-, community- and macro-level factors associated with SSB availability and intake. Over time, dental providers may become frustrated because patient behaviours do not change and the continued need to treat recurrent dental disease. This scenario can lead to provider attitudes and behaviours consistent with the belief that tooth decay prevention is impossible and a renewed focus on aggressively restoring decayed teeth. Theory-driven multilevel interventions have the potential to systematically address provider frustration, reduce barriers to oral health and improve oral health outcomes for Alaska Native children. Reducing SSB intake may also help improve systemic health outcomes. The following sections outline 5 levels at which future multilevel oral health interventions could be implemented to reduce SSB intake.

#### Tooth-level interventions

There are a number of tooth-level interventions. The first is pit-and-fissure sealants, which are plastic coatings applied to the grooves of molars. Sealants prevent tooth decay by blocking fermentable carbohydrates from accumulating in tooth grooves. Sealants can be delivered to children in dental offices or in school-based programmes. School-based sealant programmes focus on permanent first molars and are implemented in first grade, which is too late for high-risk children because decay is already present on permanent first molars when it is time to seal the teeth. Resin-based sealants are sensitive to moisture contamination. Thus, glass ionomer sealants should be placed immediately after the first molars erupt into the mouth, ideally before 5 years of age. Sealant retention should be verified and replaced when necessary at subsequent check-ups. There is currently no evidence to support primary tooth sealants, but investigators are evaluating the clinical benefits and cost effectiveness of sealing primary molars. The second tooth-level intervention is dental chemotherapeutics, which are bacteriostatic or bactericidal medicaments painted directly on the teeth at regular intervals. Topical fluoride is the most common chemotherapeutic used in dentistry. SSBs contain fermentable carbohydrates that are metabolised by cariogenic bacteria in the mouth. The acid by-products of bacterial carbohydrate metabolism lead to tooth surface demineralisation. If the process is not reversed by remineralising the tooth surface with topical fluorides, the end result is a cavity that requires treatment (e.g. filling, crown, extraction). Various topical fluoride modalities are available over-the-counter or from a health care provider (e.g. toothpaste, rinse, gel, drops, tablets, varnish). Recent evidence suggests that professionally applied topical fluoride is effective if applied at least 4 times per year (36). Other chemotherapeutics used in dentistry that interfere with bacterial adhesion, growth and/or metabolism include: povidone iodine, xylitol, diammine silver fluoride and casein phosphopeptide and amorphous calcium phosphate (CPP–ACP). Findings from the systematic review suggest that chemotherapeutics such as chlorhexidine and xylitol gum are not acceptable by Alaska Native women during pregnancy. Additional research is needed to evaluate the feasibility, acceptability, efficacy and effectiveness of chemotherapeutic interventions in Alaska Native children.

#### Patient-level interventions

There are 3 patient-level interventions that involve changing patient behaviours: (a) dietary modification; (b) oral hygiene; and (c) dental visits. Methods such as motivational interviewing have been used to change behaviours and improve patient oral health (37–40). Future dietary interventions should focus on educating patients about the benefits of reducing the frequency of SSB intake and encouraging patients to replace SSBs with water or zero-calorie beverages. Tooth brushing with fluoride toothpaste is the most effective preventive behaviour (41) because the fluoride toothpaste helps to remineralise damaged tooth structure. The effectiveness of tooth brushing is based on a number of factors (e.g. whether the toothpaste contains fluoride, amount of toothpaste used, whether an adult is helping to brush or supervising during tooth brushing, frequency of tooth brushing, how long the teeth are brushed, associated behaviours such as sharing toothbrushes or eating after tooth brushing). Tooth brushing interventions should assess a patient's current tooth brushing-related knowledge and routines, teach and enforce proper techniques and assess changes over time to track behavioural adherence and improvements in oral health. Dental visits give patients the opportunity to benefit from direct preventive care in the form of sealants and topical fluoride applications. In addition, dental visits are a time when anticipatory guidance and behavioural interventions related to diet and tooth brushing can be tailored to patients depending on caries risk level. Dietary and hygiene interventions should be firmly rooted in evidence-based behaviour change theories and implemented by trained personnel. Such interventions could be implemented by auxiliary dental providers (e.g. PDHAs) in dental offices and other settings such as medical clinics, hospitals, daycares, recreational centres, schools and churches.

#### Family-level interventions

Caregiver oral health is closely tied to children's oral health. Bacteria that cause tooth decay can be transmitted from caregiver-to-child (vertical transmission of disease) and from child-to-child (horizontal transmission of disease), which underscores the importance of preventing dental disease within families by reinforcing positive health behaviours. The same tooth- and patient-level interventions that address the consequences of SSB intake could be implemented at the family level. Educational interventions in Alaska Native communities should focus on primary caregivers within households (typically the elder matriarchs). Caregivers can lead family-based efforts to reduce SSB intake (e.g. replacing SSBs with zero-calorie beverages and water), prepare healthy meals and snacks and enforce regular tooth brushing with fluoridated toothpaste. Barriers to family tooth brushing habits could be reduced by mailing families free toothbrushes and toothpaste at regular intervals (42).

#### Community-level interventions

There are a number of community-level interventions. School boards could eliminate “pouring rights”, which are contracts between SSB companies and school districts that allow SSB companies to place vending machines on school property and advertise on sports jerseys and scoreboards in exchange for sports equipment and donations (43–45). Another option is to enact SSB taxes. Previous work has examined health outcomes associated with soda taxes (46–48) and there are preliminary discussions about enacting soda taxes in Alaska Native communities. The collected funds could be used to support activities and programmes aimed at further reducing SSB intake. Healthy Stores is an example of a community-level intervention (49–51) that involves working with store owners to encourage the sale of healthy foods and beverages, which promotes healthy purchasing by consumers. Partnerships with store owners are essential and a community-based participatory research approach can ensure cooperation and sustainability. In rural Alaska, community leaders may need to work with regional food suppliers to ensure that healthy food and beverage options are labelled and promoted and that supply channels exist year round. Other community-level interventions include water fluoridation, one of the only community-level strategies for which there are data to support effectiveness at reducing tooth decay in Alaska Native communities (14). Water fluoridation can be a politically divisive issue because of concerns raised by antifluoridationist groups (52). In addition, there are special circumstances in Alaska stemming from the Hooper Bay incident in which there was a death attributed to excess fluoride placed in the water (52). Following this incident, new regulations were enacted requiring communities to employ certified water operators and to test water fluoride levels every day. These new regulations forced many Alaska Native communities to discontinue water fluoridation programmes. Fluoridation interventions require educational outreach to community members and health providers by a local advocate or champion as well as reassurance that fluoridation is monitored by a trained water operator. In regions where there is no community water source, schools and daycares could implement fluoridated milk programmes. Promoting water consumption is a promising intervention with demonstrated effectiveness at reducing SSB intake (53–55).

#### Macro environment-level interventions

There are a number of macro-level strategies. The first is policy-level reform of the Supplemental Nutritional Assistance Program (SNAP), formerly known as the food stamp programme. Currently, there are few restrictions on the types of beverages that can be purchased by SNAP beneficiaries. Other programmes, such as the Women, Infant, and Children (WIC) Programs distribute SSBs and juice, both of which are associated with tooth decay in young children. Nationwide reforms to SNAP and WIC are likely to be met with resistance by patient advocacy groups on the grounds of governmental interference with individual choice. An alternative grassroots strategy is to generate political interest and support through parent advocacy groups. Demonstration projects with rigorous outcome evaluations could help generate the needed evidence to support larger scale policies aimed at reducing access to SSBs. Another programme that affects Alaska is the postal policy that subsidises air freight delivery of foods and beverages to remote communities in Alaska. Transportation subsidies reduce SSB costs, making SSB more accessible to children and families. In 2010, the programme cost the US Postal Service $73 million (56). Economic pressure to reduce budgets may present public health opportunities to reduce and eventually eliminate subsidies for transporting SSBs to rural Alaskan communities.

#### Additional considerations

Multilevel strategies to reduce SSB intake are resource intensive and require investments in research infrastructure, staff training, community outreach and recurring programme-related costs (e.g. chemotherapeutics, staff to deliver preventive treatments, free distribution of fluoridated toothpaste and toothbrushes, fluoridated water and milk). Despite scare resources in public health, multilevel interventions are likely to yield long-term benefits in terms of reducing childrens’ need for invasive, costly dental treatment. Based on a common risk factors approach (57), multilevel interventions aimed at reducing SSB intake are likely to also reduce systemic diseases (e.g. obesity, diabetes) among children and adult community members. Previous interventions have addressed multiple health outcomes measures, including oral health, in American Indian children (58, 59) and require consolidated data collection protocols to document and monitor changes (60).

## Conclusions

The research community has a moral obligation to address oral health disparities in Alaska Native communities. Oral health problems start in childhood and manifest throughout the adult life course. These factors provide increasing support for the development, implementation and evaluation of multilevel oral health interventions focusing on oral disease prevention in Alaska Native children and adolescents. Findings from the qualitative systematic review reveal that recent studies on oral health interventions within Alaska Native communities have focused on the training and deployment of mid-level dental providers such as the DHATs. Other types of interventions have not been documented. A case study outlined various multilevel strategies that can be used to reduce SSB intake, which is likely to improve oral and systemic health outcomes for Alaska Native children. The broader societal benefits associated with comprehensive multilevel interventions must be taken into consideration before rejecting these approaches based on high costs or perceptions that oral health interventions are of limited value. Future investigators should take advantage of the collaborative opportunities to conduct high-quality, theory-driven research on multilevel oral health interventions that bring together and build on the experiences of concerned Alaska Native community members, medical and dental health providers, public health officials and researchers.
